# Mediating Effects of Illness Acceptance and Family Intimacy on Sleep Quality and Depression in Patients With Lumbar Disc Herniation

**DOI:** 10.62641/aep.v53i5.1978

**Published:** 2025-10-05

**Authors:** Chunyan Pan, Tianyi Wu, Jun Zou, Yufang Su

**Affiliations:** ^1^Department of Orthopaedics, The First Affiliated Hospital of Soochow University, 215000 Suzhou, Jiangsu, China

**Keywords:** lumbar disc herniation, mediating effect, depression, sleep quality, illness acceptance, family intimacy

## Abstract

**Background:**

Lumbar disc herniation (LDH) is a prevalent degenerative spinal disorder, often accompanied by impaired sleep quality and depressive symptoms, which considerably affect patients’ quality of life and hinder the recovery process.

**Objective:**

This study aimed to explore the chain mediating effects of illness acceptance and family intimacy on the relationship between sleep quality and depressive symptoms in patients with LDH. The objective was to elucidate the psychosocial mechanisms influencing the psychological well-being of these patients.

**Methods:**

A total of 110 patients diagnosed with LDH between January 2022 and January 2024 were enrolled in this cross-sectional study. Participants completed validated questionnaires assessing sleep quality, illness acceptance, family intimacy and depressive symptoms, including the Pittsburgh Sleep Quality Index (PSQI), Acceptance of Illness Scale (AIS), Family Adaptability and Cohesion Evaluation Scales II (FACES II) and Self-Rating Depression Scale (SDS). Associations among the measured variables were assessed through Pearson correlation analysis. Mediation effects were analysed using the PROCESS v4.2 macro in SPSS, and the significance of the mediating effects was assessed via bootstrapping.

**Results:**

A significant positive correlation was observed between the degree of sleep disturbance and depressive symptoms (r = 0.417, *p* < 0.01). Illness acceptance and family intimacy served as key mediators in the relationship between sleep quality and depressive symptoms. Specifically, poor sleep quality was associated with increased depressive symptoms through its negative effects on family intimacy (β = –0.211, *p* = 0.027) and illness acceptance (β = –0.266, *p* = 0.003). Mediation analysis indicated that the total indirect effect accounted for 35.10% of the total effect. The chain-mediated pathway, sleep quality → family intimacy → illness acceptance → depressive symptoms, had an effect size of 0.062 (Boot SE = 0.010, 95% CI: 0.005 to 0.043), indicating that this pathway was also valid.

**Conclusion:**

Sleep quality is significantly associated with depressive symptoms in patients with LDH. Illness acceptance and family intimacy function as key mediators in this relationship. These findings underscore the importance of psychosocial factors in the mental health of patients with LDH and provide a theoretical foundation for developing targeted psychological interventions.

## Introduction

Lumbar disc herniation (LDH) is a prevalent degenerative condition of the spine [1], commonly characterised by low back pain, radiating pain in the lower extremities and sensory-motor deficits, which can severely impair patients’ quality of life [2, 3]. With the increasing aging population and the widespread adoption of sedentary lifestyles in modern society, the incidence of LDH is rising markedly, emerging as a global public health concern [4]. Although advances in surgical techniques and rehabilitation medicine have contributed to considerable improvements in the management of physical symptoms, current evidence suggests that patients with LDH frequently experience comorbid psychological disorders, especially depressive symptoms and sleep disorders [5, 6]. These psychological conditions not only intensify the perception of pain but also hinder the recovery process, potentially perpetuating a detrimental cycle. Despite these findings, limited research has explored the intricate relationship between depressive symptoms and sleep quality in patients with LDH. In particular, the potential mediating roles of psychosocial factors, such as illness acceptance and family functioning, within this relationship remain insufficiently understood.

Reduced sleep quality is a common complaint among patients with LDH, and its underlying mechanisms involves multifactorial interactions [7]. Chronic pain associated with LDH may directly disrupt sleep architecture through neurophysiological pathways, resulting in difficulties initiating sleep, frequent awakenings and non-restorative sleep [8]. Additionally, pain-related psychological stress responses, such as anxiety and depression, can further aggravate sleep problems [9]. Notably, the prevalence of depressive symptoms in patients with LDH ranges from 30% to 50%, exceeding the rates observed in the general population [5, 10]. According to the bidirectional hypothesis, a mutually reinforcing relationship exists between sleep disorders and depressive symptoms: sleep problems may intensify depressive symptoms by dysregulating neural circuits involved in mood regulation, and depression may in turn disrupts sleep by affecting the hypothalamic–pituitary–adrenal (HPA) axis [11]. Although this association has been substantiated in other chronic disease populations, the psychosocial factors that may modulate this relationship in individuals with LDH remain inadequately examined and warrant systematic investigation.

Illness acceptance has gained increasing attention in recent years as a key indicator of psychological adjustment in individuals with chronic illnesses [12]. Patients with high levels of illness acceptance generally demonstrate better adherence to treatment regimens and more adaptive coping strategies, whereas low illness acceptance is strongly associated with psychological distress and functional disability [13]. However, no study to date has directly examined the mediating role of illness acceptance in the relationship between sleep quality and depressive symptoms in patients with LDH.

Family intimacy, a crucial dimension of perceived social support, plays a key role in the management of chronic diseases [14]. Families characterised by strong emotional bonds are more likely to provide effective emotional support and practical assistance, mitigating the psychological burden of illness. In the context of LDH, family members’ support with daily functional activities, such as aiding in positional changes, can help reduce the frequency of nocturnal pain episodes, and emotional empathy may alleviate patients’ feelings of isolation and hopelessness. However, existing research has largely focused on objective measures of family support, such as caregiving time or frequency of assistance, and relatively little attention has been given to subjectively perceived family closeness and its underlying psychological mechanisms. Moreover, quantitative analyses assessing its potential mediating effects remain scarce.

Although prior studies have independently explored sleep quality, depressive symptoms, illness acceptance and family intimacy in LDH populations, the interrelationships among these variables have not been comprehensively investigated. Drawing on these theoretical foundations, we hypothesised that illness acceptance and family intimacy function as mediators in the association between sleep quality and depressive symptoms. Specifically, we propose a chain mediation model in which poor sleep quality diminishes family intimacy and illness acceptance and thereby exacerbates depressive symptoms. We aim to explore these pathways to inform the development of targeted psychosocial interventions. By enhancing illness acceptance and family intimacy, we attempted to disrupt the vicious cycle linking sleep disorders and depressive symptoms, ultimately improving quality of life and facilitating the recovery of patients with LDH.

## Materials and Methods

### Study Population

This study employed a cross-sectional survey design to investigate the relationships among illness acceptance, family intimacy, sleep quality and depressive symptoms in patients with LDH. A total of 110 eligible patients diagnosed with LDH and treated at our hospital between January 2022 and January 2024 were recruited. Ethical approval was obtained from the hospital’s ethics committee (Approval No. 2024692), and all participants provided written informed consent prior to enrolment.

The inclusion criteria were as follows: (1) age of ≥18 years and the ability to comprehend and complete the questionnaires independently; (2) diagnosis of LDH confirmed through clinical evaluation and imaging techniques, such as lumbar spine X-ray, computed tomography and magnetic resonance imaging; (3) intact cognitive function and the ability to communicate effectively; (4) disease duration of ≥3 months, ensuring that participants had undergone a period of cognitive and emotional adjustment to the condition.

The exclusion criteria were as follows: (1) comorbid serious spinal conditions (e.g., lumbar vertebral fractures, lumbar spondylolisthesis, spinal tumour); (2) comorbid systemic diseases affecting the musculoskeletal system (e.g., rheumatoid arthritis, ankylosing spondylitis); (3) diagnosed psychiatric disorders (e.g., schizophrenia, bipolar disorder) or cognitive impairments (e.g., dementia); (4) presence of serious physical illnesses (e.g., malignancies, severe cardiovascular disease) likely to impact psychological well-being; (5) history of major surgery or traumatic intervention within the preceding three months; (6) previous diagnosis of depression or other psychiatric disorders requiring ongoing antidepressant treatment.

### Data Collection

Data were collected by a team of uniformly trained medical staff during patients’ outpatient visits or periods of hospitalisation. Prior to participation, the researchers provided each patient with a detailed explanation of the study’s purpose, procedures and methodology. After obtaining written informed consent, patients completed the survey through face-to-face interviews facilitated by the data collection team. During the administration of the questionnaires, efforts were made to ensure that participants fully understood each item. In cases of ambiguity or misunderstanding, researchers offered neutral clarifications while strictly avoiding suggestive or leading prompts. Upon the completion of the questionnaires, responses were immediately reviewed for completeness and accuracy. Any missing or inconsistent data were clarified and corrected on the spot in consultation with the participant. The collected data included demographic and clinical information, such as age, gender, educational background, marital status, occupation, place of residence, primary caregiver, duration of LDH, affected spinal segment, number of acute pain episodes, treatment modalities and comorbid conditions. In addition, psychological and social variables were assessed using standardised instruments. Specifically, illness acceptance, family intimacy, sleep quality and depressive symptoms were measured using validated scales.

### Questionnaire Scale

The Acceptance of Illness Scale (AIS) was used to assess patients’ acceptance of their diagnosis of LDH [15]. Originally developed by Felton *et al*. [15], the scale was adapted and validated in Chinese by Zhao [16], yielding a Cronbach’s alpha coefficient of 0.75. The AIS comprises 8 items that evaluate patients’ attitudes towards their illness and their perceptions of its impact on daily life. Each item is rated on a 5-point Likert scale ranging from 1 (‘not at all’) to 5 (‘completely’), and higher scores indicates greater illness acceptance. In the present study, the AIS demonstrated high internal consistency, with a Cronbach’s alpha coefficient of 0.88. The intimacy subscale of the Family Adaptability and Cohesion Evaluation Scales II (FACES II) was employed to assess perceived emotional closeness within the family [17]. This subscale contains 16 items that examine emotional expression, mutual support and the quality of interpersonal relationships among family members. Responses are scored on a 5-point Likert scale, with total scores ranging from 16 to 80. Higher scores indicate greater family intimacy. The internal consistency of the scale in this study was satisfactory, with a Cronbach’s alpha coefficient of 0.82. The Pittsburgh Sleep Quality Index (PSQI) was used to evaluate patients’ sleep quality over the past month [18]. Developed by Buysse *et al*. [18] and translated into Chinese by Liu *et al*. [19], the PSQI consists of 7 components: sleep latency, duration, efficiency, disturbances and daytime dysfunction. It comprises 19 self-rated items and five additional items rated by bed partners or roommates (not scored). Each component is scored from 0 to 3, and the total PSQI score ranges from 0 to 21. Higher scores reflect poorer sleep quality. The PSQI demonstrated high internal reliability in this study, with a Cronbach’s alpha coefficient of 0.89. Depressive symptoms were assessed using the Self-Rating Depression Scale (SDS), developed by Zung [20]. The SDS consists of 20 items, each rated on a 4-point scale, from 1 (‘occasional or none’) to 4 (‘persistent or severe’). The raw total score ranges from 20 to 80, and a standard score ≥53 is indicative of clinically significant depressive symptoms. In this study, the scale showed good internal consistency, with a Cronbach’s alpha coefficient of 0.84.

### Statistical Analysis

All statistical analyses were performed using SPSS version 26.0 (IBM Corporation, Armonk, NY, USA). Continuous variables were expressed as mean ± standard deviation ($\bar{x}$ ± s), while categorical variables were presented as frequencies and percentages (n, %). Differences between two groups were assessed using the independent samples *t*-test, whereas comparisons of more than two groups were conducted using one-way analysis of variance. Correlations among the scores on each scale were assessed through Pearson correlation analysis.

To test the hypothesised mediation model, we employed Model 6 of the PROCESS macro (version 4.2) for SPSS. In this model, sleep quality was designated as the independent variable, depressive symptoms as the dependent variable, and illness acceptance and family intimacy as sequential mediators. Model 6 is specifically designed to assess chain mediation, allowing for the evaluation of serial indirect effects where one mediator (e.g., family intimacy) influences another (e.g., illness acceptance), which subsequently affects the outcome (e.g., depressive symptoms). This model is particularly well-suited to our hypothesis that the impact of sleep quality on depressive symptoms is mediated through a sequential pathway involving family intimacy and illness acceptance.

To evaluate the statistical significance of the mediating effects, we employed a bias-corrected percentile bootstrap method with 5000 resamples, generating 95% confidence intervals (CI). A mediating effect was considered statistically significant if the 95% CI did not include zero. The total effect of sleep quality on depressive symptoms, the direct effect after controlling for the mediators, and the total indirect effect were all calculated. Furthermore, specific indirect effects for each pathway were assessed, along with the proportion of the total effect accounted for by the indirect pathways. All statistical tests were two-tailed, and a *p*-value < 0.05 was considered indicative of statistical significance.

## Results

### General Characteristics and SDS Score Distribution

A total of 110 patients were included in this study, of which 47 (42.73%) and 63 (57.27%) were aged <40 and ≥40, respectively (Table 1). The patients were composed of 59 (53.64%) male patients and 51 (46.36%) female patients. No significant difference in SDS scores were found among the subgroups (*p* > 0.05), and 37 patients had SDS scores exceeding the critical value. The detection rate of depression was 33.64%.

**Table 1.** **General characteristics and SDS score distribution**.

Variable		n (%)	SDS, Mean ± *SD*	Statistic^a^	*p*
Age				*t* = −0.01	0.993
	<40	47 (42.73)	48.68 ± 9.80		
	≥40	63 (57.27)	48.70 ± 9.76		
Gender				*t* = −1.39	0.168
	Male	59 (53.64)	47.53 ± 11.01		
	Female	51 (46.36)	50.04 ± 7.90		
Degree of education				F = 0.09	0.913
	Junior high school and below	48 (43.64)	49.02 ± 8.98		
	High school or vocational school	35 (31.82)	48.11 ± 10.10		
	College degree or above	27 (24.55)	48.85 ± 10.82		
Marital status				*t* = −0.13	0.896
	Married	102 (92.73)	48.66 ± 9.97		
	Unmarried/divorced/widowed	8 (7.27)	49.12 ± 6.31		
Occupation				*t* = 0.50	0.621
	Employed	62 (56.36)	49.10 ± 9.24		
	Unemployed	48 (43.64)	48.17 ± 10.41		
Residence				*t* = 0.39	0.697
	Urban	69 (62.73)	48.97 ± 9.68		
	Suburban or rural	41 (37.27)	48.22 ± 9.91		
Minder				*t* = −0.07	0.946
	Children	92 (83.64)	48.66 ± 9.40		
	Others	18 (16.36)	48.83 ± 11.58		
LDH course				*t* = −0.31	0.754
	<12 months	58 (52.73)	48.41 ± 10.29		
	≥12 months	52 (47.27)	49.00 ± 9.17		
Diseased region				F = 0.30	0.739
	L3–4	18 (16.36)	50.33 ± 11.31		
	L4–5	44 (40.00)	48.34 ± 7.61		
	L5–S1	48 (43.64)	48.40 ± 10.92		
Number of acute pain episodes				*t* = 1.36	0.176
	<3 times	61 (55.45)	49.82 ± 9.47		
	≥3 times	49 (44.55)	47.29 ± 9.97		
Treatment				*t* = −0.88	0.380
	Surgical	71 (64.55)	48.08 ± 10.32		
	Non-surgical	39 (35.45)	49.79 ± 8.58		
Complication				*t* = 0.67	0.507
	Yes	42 (38.18)	49.43 ± 8.06		
	No	68 (61.82)	48.24 ± 10.67		

Abbreviation: LDH, lumbar disc herniation; SD, standard deviation. ^a^t, Independent sample *t*-test; F, analysis of variance.

### Scores and Correlation Analyses for Each Scale

The mean scores for PSQI, AIS, FACES II and SDS were 10.70 ± 3.48, 20.26 ± 4.01, 61.19 ± 8.28 and 48.69 ± 9.73, respectively (Table 2). Pearson correlation analysis showed that PSQI scores were significantly negatively correlated with AIS scores (r = –0.343, *p* < 0.01) and FACES II scores (r = –0.211, *p* < 0.01) and positively correlated with SDS scores (r = 0.417, *p* < 0.01). AIS scores were positively correlated with FACES II scores (r = 0.416, *p* < 0.01) and negatively correlated with SDS scores (r = –0.476, *p* < 0.01). FACES II scores were also negatively correlated with SDS scores (r = –0.400, *p* < 0.01).

**Table 2.** **Scores of each scale and correlation analysis**.

Scale	Score	Correlation coefficient			
		PSQI	AIS	FACES II	SDS
PSQI	10.70 ± 3.48	1			
AIS	20.26 ± 4.01	−0.343**	1		
FACES II	61.19 ± 8.28	−0.211**	0.416**	1	
SDS	48.69 ± 9.73	0.417**	−0.476**	−0.400**	1

**p < 0.01. Abbreviation: PSQI, Pittsburgh Sleep Quality Index; AIS, Acceptance of Illness Scale; FACES II, Family Adaptability and Cohesion Evaluation Scales II; SDS, Self-Rating Depression Scale.

### Multiple Linear Regression Analysis of the Relationship Between Variables in the Chain Mediation Model 

The PROCESS v4.2 macro in SPSS 27.0 was used to examine the chain mediating effect of illness acceptance and family intimacy on the relationship between sleep quality and depressive symptoms in patients with LDH. The results are presented in Table 3. When PSQI scores were used as the independent variable and FACES II scores as the dependent variable, PSQI had a significant negative effect on FACES II (β = –0.503, 95% CI: –0.947 to –0.060, *p* < 0.05). When AIS scores were used as the dependent variable, both PSQI (β = –0.307, 95% CI: –0.504 to –0.110, *p* < 0.01) and FACES II (β = 0.174, 95% CI: 0.092 to 0.257, *p* < 0.01) showed significant effects.

**Table 3.** **Model testing of mediation effects**.

Regression Equation		Overall Fit Indices			Regression Coefficient Significance	
Outcome variable	Predictor variable	*R* ^2^	Adjusted *R*^2^	*F*	β (95% *CI*)	*t*
FACES II	PSQI	0.045	0.036	5.056	−0.503 (−0.947 to −0.060)	−2.248*
AIS	PSQI	0.241	0.227	17.005	−0.307 (−0.504 to −0.110)	−3.092**
	FACES II				0.174 (0.092 to 0.257)	4.179**
SDS	PSQI	0.174	0.167	22.774	1.168 (0.683 to 1.654)	4.772**
SDS	PSQI	0.340	0.321	18.193	0.759 (0.291 to 1.226)	3.215**
	FACES II				−0.261 (−0.464 to −0.058)	−2.548*
	AIS				−0.705 (−1.141 to −0.269)	−3.205**

*p < 0.05; **p < 0.01. Abbreviation: PSQI, Pittsburgh Sleep Quality Index; AIS, Acceptance of Illness Scale; FACES II, Family Adaptability and Cohesion Evaluation Scales II; SDS, Self-Rating Depression Scale; CI, confidence interval.

Two regression models were established, with SDS scores as the dependent variables. (1) PSQI alone as the independent variable (β = 1.168, 95% CI: 0.683 to 1.654, *p* < 0.01); (2) PSQI (β = 0.759, 95% CI: 0.291 to 1.226, *p* < 0.01), FACES II (β = –0.261, 95% CI: –0.464 to –0.058, *p* < 0.05) and AIS (β = –0.705, 95% CI: –1.141 to –0.269, *p* < 0.01) all entered as independent variables.

### Construction of the Chain Mediation Model

**Fig. 1.**
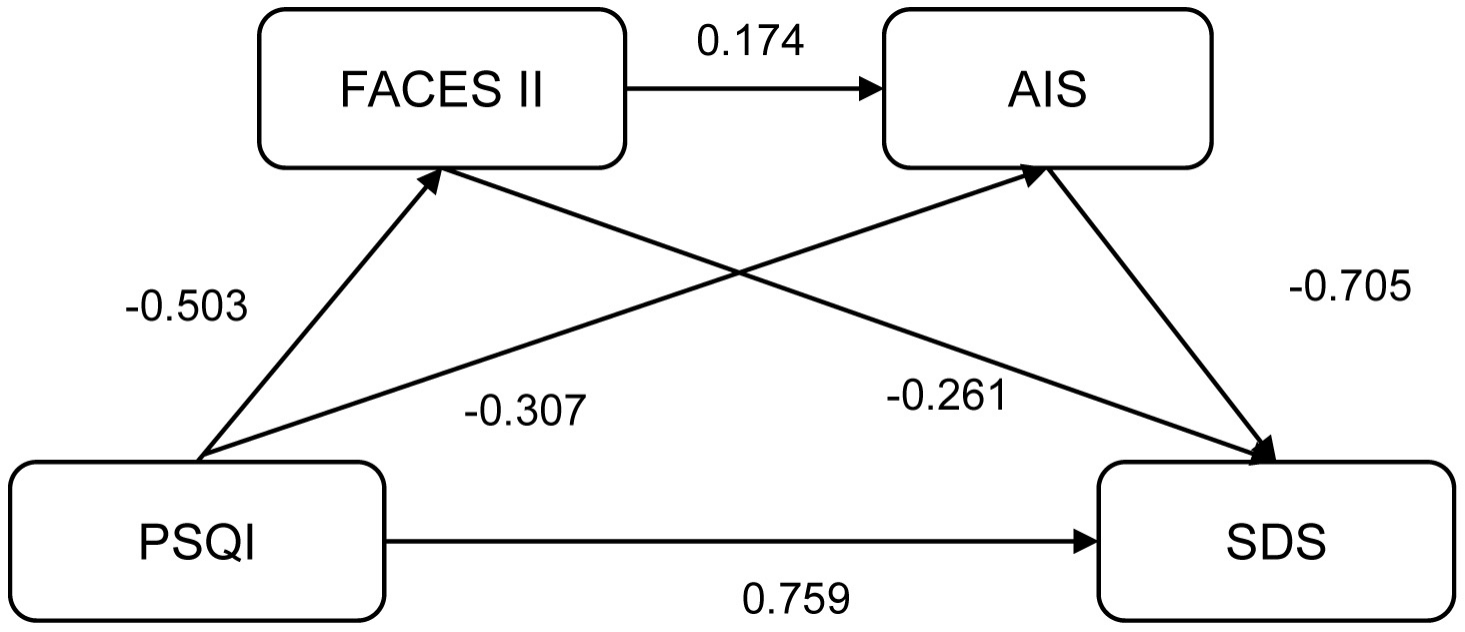
**Chain-mediated effect model of illness acceptance and family intimacy on sleep quality and depression**. Abbreviation: PSQI, Pittsburgh Sleep Quality Index; AIS, Acceptance of Illness Scale; FACES II, Family Adaptability and Cohesion Evaluation Scales II; SDS, Self-Rating Depression Scale.

The chain mediating role of illness acceptance and family intimacy in the relationship between sleep quality and depressive symptoms was assessed using Model 6 of the PROCESS v4.2 macro (Fig. 1). As shown in Table 4, the total effect of sleep quality on depressive symptoms was 1.168 (95% CI: 0.688 to 1.648, *p* < 0.001). After the mediating variables were included, the direct effect was 0.759 (95% CI: 0.296 to 1.221, *p* = 0.002), accounting for 64.98% of the total effect. The total indirect effect was 0.410 (95% CI: 0.064 to 0.240, *p* < 0.001), accounting for 35.02% of the total effect.

**Table 4.** **Summary of effect analysis process**.

Effect	Term	Effect	*SE*	*t*/*z*	*p*	LLCI	ULCI	Effect proportion
Total effect	Sleep → depression	1.168	0.245	4.772	<0.001	0.688	1.648	100.00%
Direct effect	Sleep → depression	0.759	0.236	3.215	0.002	0.296	1.221	64.98%
Total indirect effects	Sleep → depression	0.410	0.045	9.105	<0.001	0.064	0.240	35.02%

Abbreviation: *SE*, standard error; LLCI, bootstrap sampling lower 95% interval limit; ULCI, bootstrap sampling upper 95% interval limit.

### Tests of the Results of the Mediation Model

The bootstrap method was used to test the significance of the indirect effects and verify the presence of mediation. As shown in Table 5, the indirect effect of the pathway sleep → family intimacy → depression was 0.131 (95% CI: 0.005 to 0.113, *p* < 0.001), accounting for 11.22% of the total effect. The indirect effect of the pathway sleep → illness acceptance → depression was 0.217 (95% CI: 0.026 to 0.141, *p* < 0.001), also accounting for 18.58% of the total effect. The chain-mediated pathway sleep → family intimacy → illness acceptance → depression had an effect size of 0.062 (95% CI: 0.005 to 0.043, *p* < 0.001), representing 5.31% of the total effect.

**Table 5.** **Indirect effect path analysis**.

Term	Effect	*SE*	LLCI	ULCI	*z*	*p*	Effect proportion
Sleep → family intimacy → depression	0.131	0.028	0.005	0.113	4.670	<0.001	11.22%
Sleep → illness acceptance → depression	0.217	0.029	0.026	0.141	7.355	<0.001	18.58%
Sleep → family intimacy → illness acceptance → depression	0.062	0.010	0.005	0.043	6.286	<0.001	5.31%

Abbreviation: *SE*, standard error; LLCI, bootstrap sampling lower 95% interval limit; ULCI, bootstrap sampling upper 95% interval limit.

## Discussion

This study demonstrates the considerable chain-mediating roles of illness acceptance and family intimacy in the relationship between sleep quality and depressive symptoms among patients with LDH. These findings must be interpreted within the clinical and psychosocial context specific to LDH. As a common degenerative spinal condition, LDH often results in chronic low back pain and radiating lower extremity pain, which markedly impair sleep quality and emotional well-being [21]. Compared with other chronic diseases, the persistent nociceptive input and direct involvement of neural pathways in LDH may uniquely exacerbate sleep disturbances and depressive symptoms. Additionally, the functional limitations imposed by LDH can disrupt patients’ social roles and daily activities, particularly within the family context, potentially leading to decreased family intimacy and illness acceptance. The present findings suggest that enhancing illness acceptance and strengthening family intimacy may serve as effective targets for mitigating the psychological consequences of poor sleep quality in this population. These mediating factors provide important insights into the psychosocial mechanisms underlying emotional distress in LDH and highlight the need for comprehensive, tailored psychological interventions that go beyond physical symptom management. This study thus contributes a novel theoretical framework for the development of integrated care approaches for patients with LDH.

Firstly, the results of this study indicated a significant positive correlation between PSQI and SDS scores, suggesting that poorer sleep quality is associated with more severe depressive symptoms. This finding is consistent with previous research in other chronic disease populations, supporting the well-established bidirectional relationship between sleep disturbances and depression [22, 23]. Sleep deprivation or poor sleep quality can disrupt the metabolism and regulation of key neurotransmitters involved in mood regulation, contributing to the onset or worsening of depressive symptoms [24]. However, owing to the cross-sectional design of this study, reverse causality cannot be ruled out, that is, depressive symptoms may exacerbate sleep disorders. In depressive states, abnormal brain activity and dysregulation of the HPA axis can further disrupt sleep architecture, perpetuating a vicious cycle [25, 26]. In the context of LDH, persistent pain serves as a major contributor to both sleep disruption and depressive symptoms. Continuous nociceptive input interferes with the normal sleep-wake cycle, and prolonged pain experience often leads to emotional distress, thereby increasing the risk of depression [27].

Further analysis revealed that illness acceptance serves as an essential mediating factor in the relationship between sleep quality and depressive symptoms. The findings indicate that poor sleep quality is associated with lower levels of illness acceptance, which, in turn, is linked to higher levels of depressive symptoms. From a psychological perspective, diminished sleep quality contributes to physical fatigue and discomfort, which may impair patients’ cognitive and emotional capacity to process their health status. This can hinder their ability to accept the illness and increase vulnerability to negative self-perceptions and emotional distress [28]. In contrast, individuals with higher illness acceptance are more likely to adopt adaptive coping strategies, such as adhering to medical treatment and engaging in appropriate physical activity, which contribute to better psychological outcomes and may help reduce depressive symptoms [29]. Moreover, those who accept their condition are typically better equipped to manage disease-related stress and are less prone to emotional dysregulation, which can, in turn, enhance sleep quality. Thus, promoting illness acceptance may represent a critical intervention target for improving the psychological well-being of patients with LDH.

In addition, family intimacy was identified as a key mediating variable in the relationship between sleep quality and depressive symptoms. The analysis revealed that greater sleep disturbances are associated with lower levels of family intimacy, and that reduced family intimacy is negatively correlated with depressive symptoms. The family unit serves as an important source of social support for individuals with chronic illnesses, offering emotional reassurance and practical assistance [30]. When sleep is disrupted because of disease-related symptoms, attentive care and emotional support from family members can alleviate feelings of isolation and helplessness, thereby buffering against psychological distress [31]. For instance, assistance from family members in adjusting sleeping positions at night can help minimise pain episodes and enhance sleep quality. Furthermore, higher levels of family intimacy reflect more effective communication, mutual understanding and emotional responsiveness among family members [32]. This supportive family environment fosters psychological resilience, helping patients maintain a stable emotional state and reducing their susceptibility to depressive symptoms. Notably, the findings also suggest that family intimacy indirectly mitigates depression by enhancing illness acceptance, forming a chain mediating pathway of ‘sleep quality → family intimacy → illness acceptance → depression’. This highlights the central role of the family system in the psychosocial management of chronic diseases such as LDH [33]. These results are consistent with family systems theory, which posits that strong familial bonds can enhance patients’ self-efficacy and sense of belonging, ultimately promoting more adaptive responses to illness [34].

However, this study has several limitations. First, the cross-sectional design precludes any inference of causality among the observed variables, limiting the findings to correlations rather than directional relationships. Future research should employ longitudinal designs to monitor changes in patients’ psychological and physiological states over time, thereby providing stronger evidence for causal mechanisms. Second, the limited sample size and recruitment from a single or few clinical centers may reduce the representativeness of the findings. Owing to sample constraints, this study was also unable to control for certain important covariates, such as age, gender and comorbid conditions. Future studies should expand the sample to include patients from diverse geographic regions and healthcare settings to enhance generalizability and enable stratified analyses. Third, this study explored only the mediating effects of illness acceptance and family intimacy and did not consider other factors that may affect the relationship between sleep quality and depressive symptoms. Mechanistic analyses should be examined in future models using more comprehensive assessment tools. Finally, the R^2^ value in the current chain mediation model was relatively modest, indicating the presence of unexplored confounding factors (such as pain catastrophisation and inflammation levels). Nevertheless, the observed mediating effects are statistically and clinically meaningful. In clinical research, even a moderate effect size can be impactful when it informs actionable interventions for at-risk populations. Despite these limitations, our findings clarify key psychosocial mechanisms linking sleep quality and depression in patients with LDH, offering practical implications for clinical management: The strong association between sleep quality and depressive symptoms, alongside the high prevalence of depressive symptoms observed in this study (33.64%), underscores the need for routine screening of both domains in patients with LDH. Clinicians should incorporate validated tools such as the PSQI and SDS during initial evaluations, particularly for patients reporting sleep issues. A brief combined assessment (approximately 5–10 minutes) in outpatient clinics could help identify individuals at high risk of depression early, especially those exhibiting poor sleep and low family intimacy. Given the mediating role of illness acceptance, healthcare providers should develop structured psychological interventions aimed at enhancing patients’ acceptance of their condition. For instance, cognitive-behavioural therapy programs could include weekly group sessions focused on cognitive restructuring, where patients reframe catastrophic thoughts about LDH into manageable narratives. These programs can be supplemented by skill-building exercises, such as practicing ergonomic movements or journaling progress, to foster a sense of control and self-efficacy, thereby reducing depressive symptoms. The identification of a chain-mediating effect involving family intimacy highlights the importance of engaging family members in treatment plans. Hospitals can implement monthly caregiver workshops (e.g., two-hour sessions) to train family members in practical support techniques. These workshops may include assisting with nocturnal position adjustments to reduce pain-induced sleep disruptions, employing active listening to enhance emotional connection, and recognising early behavioural signs of depression (e.g., social withdrawal, irritability). Such interventions not only strengthen family cohesion but also create a long-term psychosocial buffer that supports recovery beyond clinical visits, directly addressing the ‘sleep → family intimacy → depression’ pathway.

## Conclusion

LDH is a common degenerative spinal disorder frequently associated with reduced sleep quality and depressive symptoms, which considerably impair patients’ quality of life and recovery. This cross-sectional study investigated the potential mediating roles of illness acceptance and family intimacy in the relationship between sleep quality and depressive symptoms in patients with LDH. The findings revealed a significant positive correlation between sleep disturbances and depressive symptoms. Moreover, both illness acceptance and family intimacy were found to play crucial chain-mediating roles in this association. Specifically, poor sleep quality contributed to increased depressive symptoms by diminishing patients’ acceptance of their illness and weakening familial support.

These results underscore the importance of psychosocial factors in shaping the mental health outcomes of individuals with LDH. Interventions that enhance illness acceptance and strengthen family intimacy may help disrupt the negative cycle between sleep disturbances and depression. Clinically, this study offers a novel perspective for the holistic management of LDH, highlighting the value of integrating psychosocial assessments into routine care. Tailored psychological interventions that address these mediators can provide more effective support for patients beyond physical symptom management. Additionally, the study offers directions for future research by emphasising the need to examine the interaction between psychosocial and biological mechanisms, ultimately aiming to optimise therapeutic strategies and promote comprehensive recovery in LDH populations.

## Availability of Data and Materials

The datasets generated during and/or analyzed during the current study are available from the corresponding author upon reasonable request.
